# Inhibition of Matrix Metalloproteinase with BB-94 Protects against Caerulein-Induced Pancreatitis via Modulating Neutrophil and Macrophage Activation

**DOI:** 10.1155/2020/8903610

**Published:** 2020-04-28

**Authors:** Zengkai Wu, Tunike Mulatibieke, Mengya Niu, Bin Li, Juanjuan Dai, Xin Ye, Yan He, Congying Chen, Li Wen, Guoyong Hu

**Affiliations:** ^1^Department of Gastroenterology, Shanghai General Hospital, Shanghai Jiao Tong University School of Medicine, Shanghai, China; ^2^Shanghai Key Laboratory of Pancreatic Disease, Institute of Pancreatic Disease, Shanghai Jiao Tong University School of Medicine, Shanghai, China; ^3^Department of Cadre Ward, People's Hospital of Xinjiang Uygur Autonomous Region, Xinjiang, China

## Abstract

**Methods:**

AP was induced in Balb/C mice by ten hourly intraperitoneal injections of caerulein (100 *μ*g/kg) and LPS (5 mg/kg). The MMP inhibitor, BB-94 (20 mg/kg) was intraperitoneally administered 30 min before AP induction. Pancreatitis was confirmed by histology and serum amylase and lipase. Expression of pancreatic proinflammatory mediators and NF-*κ*B activation were assessed. Bone marrow-derived neutrophils (BMDNs) and macrophages (BMDMs) were isolated. BMDNs were activated by phorbol 12-myristate 13-acetate (PMA, 50 ng/ml) and neutrophil reactive oxygen species (ROS) production was recorded. BMDMs were stimulated with 10 ng/ml IFN-*γ* and 100 ng/ml LPS to induce M1 macrophage polarization.

**Results:**

Pancreatic MMP-9 was markedly upregulated and serum MMP-9 was increased in caerulein-induced pancreatitis. Inhibition of MMP with BB-94 ameliorated pancreatic tissue damage and decreased the expression of proinflammatory cytokines (TNF*α* and IL-6) or chemokines (CCL2 and CXCL2) and NF-*κ*B activation. Furthermore, using isolated BMDNs and BMDMs, we found that inhibition of MMP with BB-94 markedly decreased neutrophil ROS production, inhibited inflammatory macrophage polarization and NF-*κ*B activation.

**Conclusions:**

Our results showed that inhibition of MMP with BB-94 protected against pancreatic inflammatory responses in caerulein-induced pancreatitis via modulating neutrophil and macrophage activation.

## 1. Introduction

Acute pancreatitis (AP) is a common and potentially life-threatening inflammatory disorder of the pancreas and is the leading cause of hospital admission for gastrointestinal disorders worldwide [1–3]. The overall mortality associated with AP has been decreasing, but the mortality among severe AP characterized by persistent organ failure and pancreatic necrosis remains as high as 30% [1]. Despite the substantial morbidity and mortality, there is still no specific treatment approved for patients with AP. Management of AP is restricted to supportive care, which is partly due to our incomplete understanding of AP pathophysiology. It has been implicated for many years that premature activation of trypsinogen within pancreatic acinar cells (PACs) initiates AP, leading to PAC injury [4, 5]. Injured PACs produce proinflammatory mediators to recruit circulating leukocytes into the pancreas, which in turn amplifies local and systemic inflammatory responses, leading to multiple organ failure [6–8].

Matrix metalloproteinases (MMPs) function as endopeptidases to cleave the majority of matrix proteins as well as many nonmatrix targets, such as chemokines, cytokines, and adhesion molecules [9–11]. Certain MMPs, particularly MMP-9, have been reported to be closely related to pancreatitis severity and distant organ damage in different experimental models of pancreatitis as well as patients with AP [12–19]. Accumulating evidence in the literature have demonstrated that inhibition of MMPs with broad-spectrum inhibitors attenuates leukocyte infiltration, systemic inflammatory responses, and tissue damages in AP [12, 14, 16–18]. However, the role and mechanism of MMP inhibition on inflammatory cell activation remains elusive. Therefore, in this study, we sought to examine inhibition of MMP-9 with BB-94 on neutrophil and macrophage activation in caerulein-induced pancreatitis.

Firstly, we found that pancreatic MMP-9 was significantly upregulated, and serum MMP-9 was also elevated in caerulein-induced pancreatitis. Inhibition of MMP with BB-94 attenuated pancreatic edema, inflammatory infiltration, necrosis, and also markedly reduced pancreatic proinflammatory cytokine or chemokine expression and NF-*κ*B activation. Moreover, using isolated bone marrow-derived neutrophils (BMDNs) and macrophages (BMDMs), we found that inhibition of MMP with BB-94 significantly reduced neutrophil reactive oxygen species (ROS) and inhibited inflammatory macrophage polarization. These findings revealed that MMPs, particularly MMP-9, play a crucial role in mediating inflammatory cell activation during AP, suggesting that specifically targeting MMP-9 could be a potential therapeutic approach for treating AP.

## 2. Methods

### 2.1. Animals

Balb/C mice (male, 6 weeks, 20-21 g) were purchased from Shanghai SLAC Laboratory Animal Co Ltd. (Shanghai, China). The animals were housed for 1 week under specific-pathogen-free conditions at a temperature of 22°C and 12 h dark/light cycle and allowed standard rodent diet and water ad libitum before experimentation. All mice (male, 21-22 g) were randomly allocated into experimental groups (*n* = 5 per group). All procedures were approved by the Animal Ethics Committee of Shanghai Jiao Tong University School of Medicine (SYXK 2013-0050, Shanghai, China).

### 2.2. Induction of Experimental Pancreatitis and Treatments

Caerulein-induced pancreatitis (CER) was used in this study, which is a noninvasive, widely used, and highly reproducible model of experimental AP [20]. CER was induced by 10 hourly intraperitoneal injections of caerulein (100 *μ*g/kg) and LPS (5 mg/kg) was intraperitoneally administered immediately after the last injection of caerulein. Mice were sacrificed humanely 12 h after the first caerulein injection. Mice received the same volume of saline served as the control. BB-94 (20 mg/kg) was intraperitoneally administered 30 min before the first injection of caerulein.

### 2.3. Serological Test

Blood samples were collected and centrifuged at 2000 rpm for 20 min at 4°C to obtain the serum. Serum amylase and lipase were measured by enzyme dynamics chemistry using commercial kits according to the manufacturer's protocols in a Roche/Hitachi modular analytics system (Roche, Switzerland). Serum MMP-9 was measured by ELISA according to the manufacturer's protocols (Westang Bio-Tech Co, LTD, Shanghai, China).

### 2.4. Hematoxylin–Eosin and Immunohistochemical Staining

Fresh specimens of the pancreas were fixed in 4% neutral paraformaldehyde for 24 h, embedded in paraffin, and 4 *μ*m sections were processed for H&E staining by standard procedures as previously described [21]. The pancreas sections were scored from 0 to 3 (0: normal and 3: severe) for edema, inflammation, and necrosis [22], respectively, by two independent experienced pathologists in a blind fashion. For immunohistochemistry staining, endogenous peroxidase was blocked by 3% hydrogen peroxide. Sections were then incubated overnight at 4°C with a monoclonal antibody against Ly6G (1 : 50, Abcam, Cambridge, UK). After rinsed in the phosphate buffer solution (PBS) for three times, sections were incubated with secondary antibody for 1 h at 37°C, then visualized by an ultrasensitive SP kit and a dopamine B kit (Maxin, Fuzhou, China) and examined under the light microscope (Leica, Wetzlar, Germany).

### 2.5. Quantitative PCR

Total RNA was extracted from pancreatic tissue and BMDMs using Trizol reagent (Invitrogen, CA, USA) as previously described [23]. cDNA samples were prepared from total RNA using SuperScript II preamplification kit (Fermentas, MD, USA) according to the manufacturer's instructions. The synthesized cDNA was then used as a template for qRT-PCR with gene-specific, intron-spanning primers listed in Table 1. Quantitative PCR (qPCR) was performed on ABI Prism 7900HT Sequence Detection System (Applied Biosystems, CA, USA) using KAPA SYBR Kits (Kapa Biosystems, Wilmington, USA). Relative expression levels of target genes were normalized to the housekeeping gene *β*-actin, and fold changes were calculated using the comparative CT (2^−ΔΔCT^) method. Each target gene was analyzed in triplicate in each triplicate experiment.

### 2.6. Western Blotting

The total protein of pancreatic tissues was extracted as previously described [21]. Protein concentrations were detected using a bicinchoninic acid protein assay kit (Beyotime Biotechnology, China). 40 *μ*g of protein samples were loaded in each lane, separated on 10% sodium dodecyl sulfate-polyacrylamide gel electrophoresis (SDS-PAGE) and transferred to nitrocellulose membranes (Millipore, USA). BSA (5%) was used for 60 min, then blots were incubated with primary antibodies against MMP-9 (1 : 500, Abcam, Cambridge, UK), TNF-*α* (1: 500, Proteintech, Rosemont, USA), IL-6 (1 : 500, Cell Signaling Technology, Boston, USA), NF-*κ*B p65 (1 : 400, Cell Signaling Technology, Boston, USA), p-NF-*κ*B p65 (1 : 400, Cell Signaling Technology, Boston, USA), I*κ*B*α* (1 : 400, Cell Signaling Technology, Boston, USA), p-I*κ*B*α* (1 : 500, Cell Signaling Technology, Boston, USA), *β*-actin (1 : 2000, Proteintech, Rosemont, USA), and *α*-actinin (1 : 1000, Cell Signaling Technology, Boston, USA) overnight at 4°C. After washing in the PBS containing 0.1% Tween three times, blots were probed with goat antirabbit or goat antimouse IR-Dye 800 or 700 CW labeled secondary antibodies for 1 h at 37°C. Signals were visualized by Odyssey infrared scanner (LI-COR, USA). The protein level in different groups was normalized to *β*-actin or *α*-actinin, and the phosphorylation level of target proteins was compared with their total level. The relative expression of target proteins was presented as fold changes compared to the control group.

### 2.7. Isolation of Bone Marrow-Derived Neutrophils and Macrophages and Cell Culture

BMDNs and BMDMs were isolated from mouse femur and tibia. Following the surgical dissection of the bones, the femur and tibia were flushed with sterile PBS (Sangon, Shanghai, China) and passed through a 70 *μ*m sterile filter (Fisher Scientific, Waltham, USA) to obtain the crude bone marrow cells. After centrifugation at 600 g for 5 minutes at 4°C with the brake off, cells were resuspended in 4 ml PBS and loaded on top of a discontinuous density gradient (62% and 81% Percoll) [24] and centrifuged at 1500 g for 20 min at 4°C with the brake off. BMDNs were collected between 62% and 81% Percoll layers and suspended in 4 ml Red Cell Lysis Buffer (eBiosciences, multispecies) for 5 minutes on a rocker, washed with PBS and centrifuged at 600 g for 5 min before resuspending in RPMI1640 (Hyclone, Logan, USA). BMDNs were counted using 0.4% trypan blue, typically >95% purity and >90% viability. BMDMs were collected on top of 62% Percoll layer and washed with PBS and centrifuged at 400 g for 5 min at 4°C with the brake on. BMDMs were then counted using 0.4% trypan blue, typically >95% purity and >90% viability, and cultured for 6 days in DMEM medium supplemented with 10% heat-inactivated FBS, 1% l-glutamine, 1% penicillin/streptomycin antibiotics, and 20 ng/mL M-CSF. After 6-day culture, BMDMs were stimulated with 100 ng/mL LPS and 10 ng/mL IFN-*γ* with or without BB-94 for 24 h. Naive macrophages (M0) were left unstimulated and served as the control.

### 2.8. Chemiluminescence Measurement of ROS Production

Neutrophil ROS production was monitored by peroxidase-enhanced luminol chemiluminescence as previously described [25], using Synergy H1 plate reader (BioTek, Winooski, Vermont, USA). Briefly, BMDNs were plated (500,000 cells per well), then added with 50 *μ*M luminol and 75 units/ml horseradish peroxidase, and stimulated with 50 ng/ml phorbol 12-myristate 13-acetate (PMA, Sigma) with or without BB-94 pre-treatment. ROS production was recorded for 30 min. The area under the curve (AUC) was calculated, normalized to negative control for each mouse/run, averaged across the runs, and converted to mean ± SEM for a minimum of 3 or more mice per experimental group.

### 2.9. Statistical Analysis

All data were presented as mean ± SEM from at least three independent experiments. Comparisons between two groups were determined by two-tailed Student's *t* test. Significant differences among three or more groups were compared using one-way ANOVA with Bonferroni's posttest. Statistical analysis was performed using GraphPad Prism version 7.0 (La Jolla, CA, USA). *p* values <0.05 were considered statistically significant.

## 3. Results

### 3.1. MMP-9 Is Upregulated in Acute Pancreatitis

**Figure 1:**
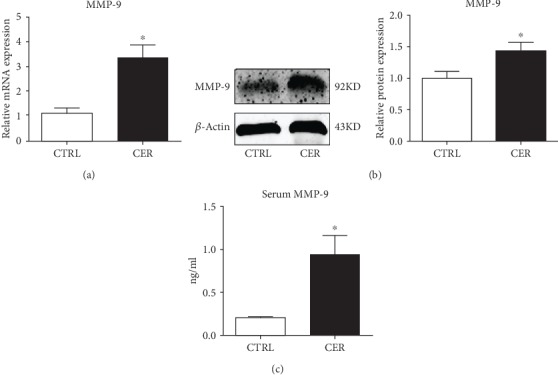
MMP-9 is upregulated during caerulein-induced pancreatitis. (a) mRNA levels of MMP-9 in the pancreas. (b) Protein levels of MMP-9 in the pancreas. (c) Serum MMP-9 levels. *n* = 5 mice per group; ∗*p* < 0.05 vs the control group.

In caerulein-induced pancreatitis, we measured pancreatic MMP-9 mRNA and protein levels by qPCR and western blotting and found that both mRNA and protein levels of MMP-9 were markedly upregulated in caerulein-induced pancreatitis (Figure 1(a) and 1(b)). Similarly, serum MMP-9 was significantly elevated (Figure 1(c)). Consistent with previously published studies [19], we showed that MMP-9 is upregulated in AP, suggesting that it may be a primary regulator in the pathogenesis of AP.

### 3.2. Inhibition of MMP with BB-94 Protects against Caerulein-Induced Pancreatitis

**Figure 2:**
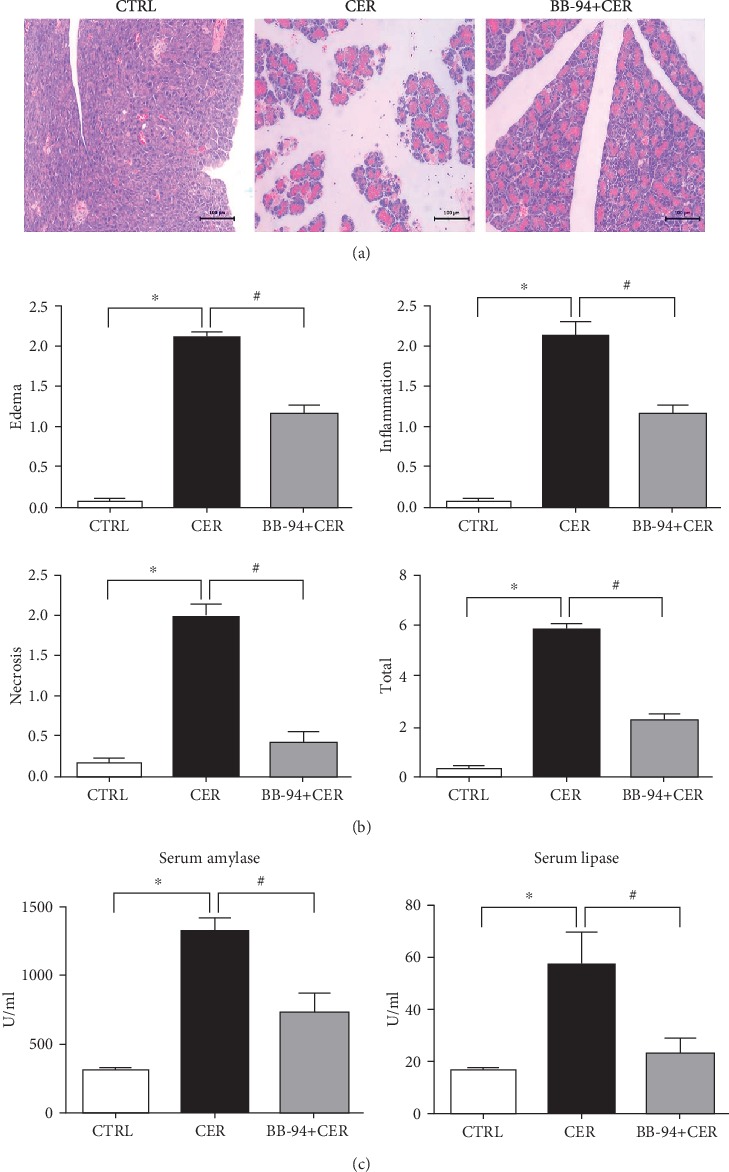
Inhibition of MMP ameliorates pancreatic histology, serum amylase, and lipase in caerulein-induced pancreatitis. (a) H&E staining of pancreatic tissue from the control, CER, and CER plus BB94. (b) Histopathological subscores for edema, inflammation, and necrosis and the total histopathological score calculated by summation the subscores. (c) Serum amylase and lipase. *n* = 5 mice per group; ∗*p* < 0.05 vs the control group; #*p* < 0.05 vs the CER group.

We next examined whether inhibition of MMP mediates pancreatic injury. MMP was inhibited by a broad-spectrum MMP inhibitor, BB-94 [12, 14, 16, 17], which is a potent inhibitor of MMP-1, 2, 3, 7, and 9. BB-94 was intraperitoneal administered 30 min before the first injection of caerulein. Pancreatic histology and serum markers were assessed 12 h after the first injection. We observed that inhibition of MMP with BB-94 markedly reduced pancreatic histology as assessed by pancreatic edema, inflammatory infiltration, and acinar cell necrosis (*p* < 0.05, Figure 2(b)). Similarly, serum amylase and lipase were significantly decreased with BB-94 (Figure 2(c)). Consistent with previous reports [14, 17], our data demonstrated that MMP inhibition with a broad-spectrum MMP inhibitor protects against the severity of caerulein-induced pancreatitis.

### 3.3. Inhibition of MMP with BB-94 Mitigates Pancreatic Inflammation

**Figure 3:**
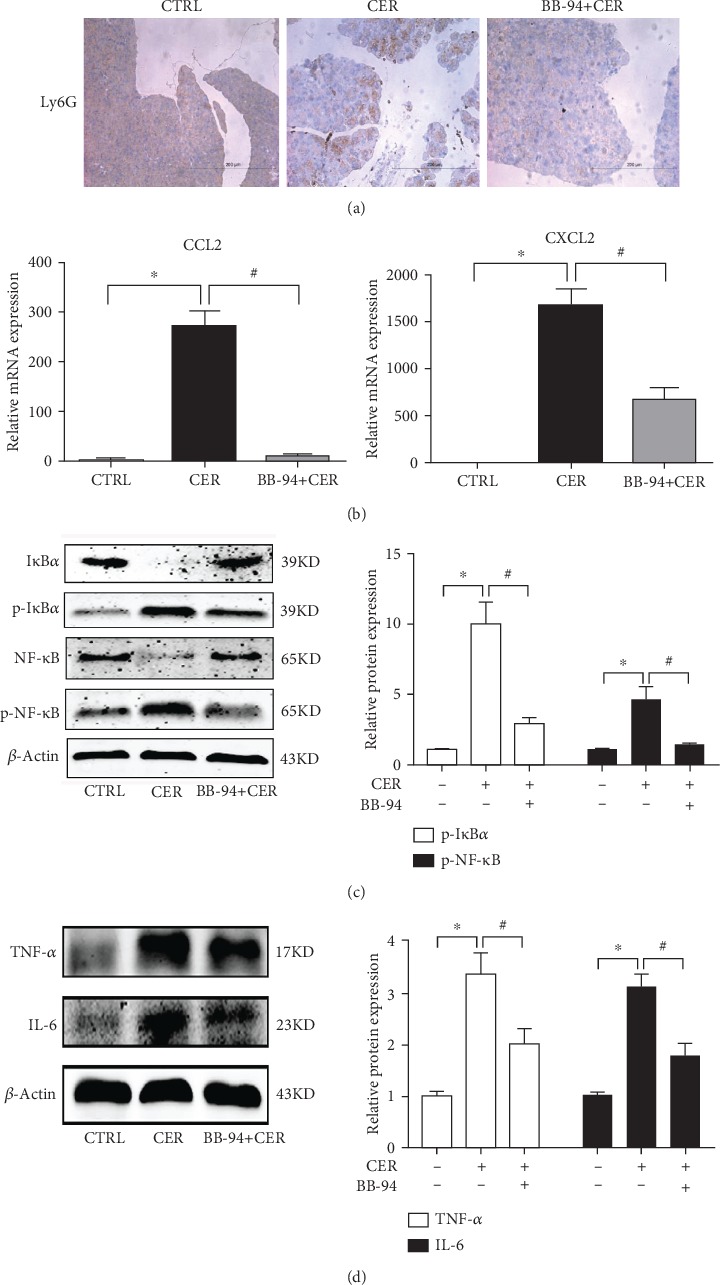
Inhibition of MMP reduces pancreatic inflammatory responses in caerulein-induced pancreatitis. (a) Immunohistochemical analysis of pancreatic immune cell infiltration, Ly6G for neutrophils. (b) mRNA levels of CCL2 and CXCL2 in the pancreas. (c) Protein levels of I*κ*B*α*, phospho-I*κ*B*α*, NF-*κ*B and phospho-NF-*κ*B in the pancreas. (d) Expression of IL-6 and IL-1*β* in the pancreas by western blot. *n* = 5 mice per group; ∗*p* < 0.05 vs the control group; #*p* < 0.05 vs the CER group.

Accumulating evidence from the previous studies suggest that a critical role of MMP-9 in mediating organ damages and inflammatory responses [12–14, 17]. We next examined the impact of BB-94 on pancreatic inflammatory responses. Immunohistochemistry staining for pancreatic tissue from control, caerulein-induced pancreatitis, and caerulein-induced pancreatitis with BB-94 revealed that MMP inhibition decreased pancreatic inflammatory infiltration stained by Ly6G (Figure 3(a)). Moreover, chemokines for neutrophil (CXCL2) and macrophage (CCL2) recruitment were also downregulated with BB-94 (Figure 3(b)). the activation of the central proinflammatory signal NF-*κ*B in the pancreas was largely inhibited by BB-94 (Figure 3(c)). Lastly, The protein levels of proinflammatory cytokines including TNF-*α* and IL-6 were significantly downregulated by BB-94 (Figure 3(d)). Taken together, these results showed that MMP inhibition significantly deceased pancreatic inflammatory responses during AP.

### 3.4. Inhibition of MMP Mediates Neutrophil and Macrophage Activation

**Figure 4:**
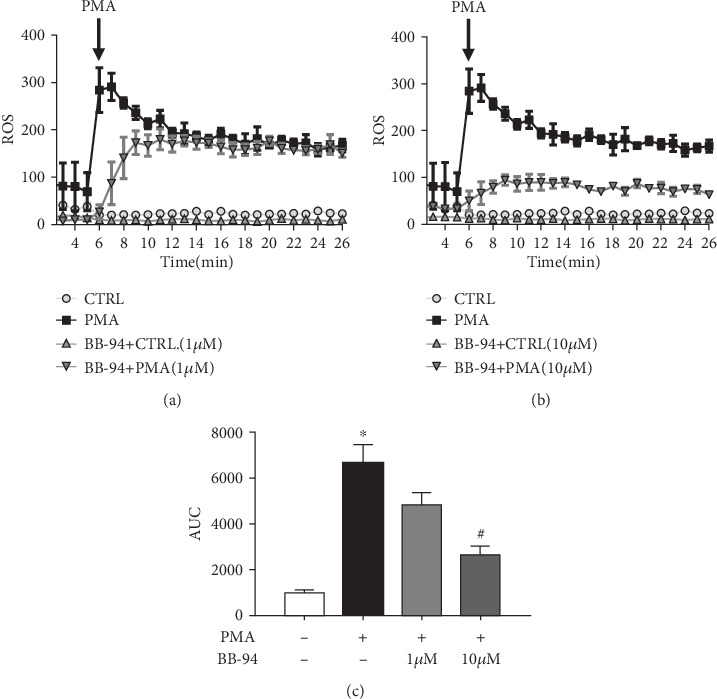
Inhibition of MMP reduces neutrophil ROS production *in vitro*. Bone marrow-derived neutrophils (BMDNs) were isolated and stimulated with phorbol 12-myristate 13-acetate (PMA, 50 ng/ml; Sigma). Total ROS production was measured and recorded for 25 min by chemiluminescence in the presence of (a) 1 *μ*M BB-94 and (b) 10 *μ*M BB-94. (c) The area under the curve (AUC) was calculated, normalized to negative control for each mouse/run. *n* = 3 BMDN isolation per conditions; ∗*p* < 0.05 vs the control group; #*p* < 0.05 vs the PMA-stimulated group.

Since inhibition of MMP markedly reduced pancreatic inflammatory infiltration, the activation of which has been implicated to play a crucial role in mediating additional tissue damage [26–28]. Next, we examined whether MMP inhibition affects neutrophil and macrophage activation. Isolated BMDNs were stimulated with PMA, a potent activator of ROS production in neutrophil [25], in the presence or absence of various concentrations of BB-94. We found that PMA-induced neutrophil ROS production was markedly reduced by BB-94 pretreatment with a more marked reduction at the higher concentration of BB-94 (*p* < 0.05, Figure 4). These findings suggest that inhibition of MMP mitigates inflammatory responses via reducing neutrophil ROS production.

**Figure 5:**
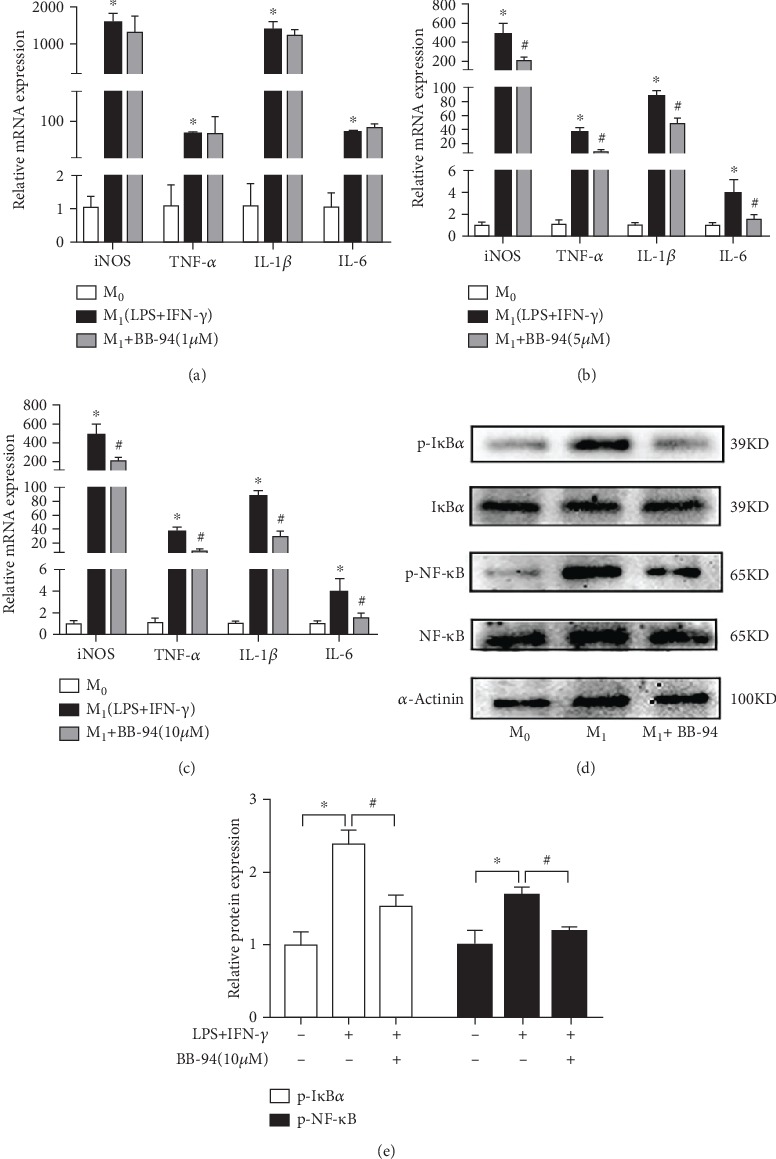
Inhibition of MMP inhibits inflammatory macrophage polarization *in vitro*. BMDMs were left untreated as naive unstimulated macrophages or stimulated using 100 ng/mL LPS and 10 ng/mL IFN-*γ* with or without BB-94 treatment for 24 h. The M1 macrophage-specific markers, including INOS, IL-1*β*, IL-6, and TNF-*α* were measured by qPCR (a) with 1 *μ*M BB-94 treatment, (b) with 5 *μ*M BB-94 treatment, and (c) with 10 *μ*M BB-94 treatment. (d and e) Expression and quantification of I*κ*B*α*, phospho-I*κ*B*α*, NF-*κ*B, and phospho-NF-*κ*B in M1-polarized macrophages by western blot. *n* = 3 BMDM isolation per conditions; ∗*p* < 0.05 vs the control group; #*p* < 0.05 vs the LPS+IFN-*γ*-stimulated group.

Classically activated macrophages (M1) are characterized by a prominent proinflammatory phenotype and play a critical role in driving tissue damage [29]. To determine the effects of BB-94 on proinflammatory macrophage polarization, BMDMs were stimulated with 100 ng/mL LPS and 10 ng/mL IFN-*γ* to induce M1 macrophage polarization in the presence or absence of various concentrations of BB-94. The expression of M1 macrophage-specific markers were examined by qPCR. Inhibition of MMP with BB-94 diminished the mRNA expression of all the inflammatory macrophage genes, including iNOS, TNF-*α*, IL-1*β*, and IL-6 (*p* < 0.05, Figures 5(a)–5(c)). These results suggest that MMP, likely MMP-9, plays a critical role in mediating inflammatory macrophage polarization. Since NF-*κ*B signaling pathway contributes to the maintenance of polarized macrophage status [30]. We next examined the impact of BB-94 on NF-*κ*B activation in M1-polarized macrophages and found that inhibition of MMP significantly downregulated the protein levels of p-I*κ*B*α* and p-NF-*κ*B p65 (Figures 5(d) and 5(e)). Collectively, these data demonstrated that inhibition of MMP prevents pancreatic inflammatory responses via inhibiting inflammatory macrophage polarization.

## 4. Discussion

In this study, we demonstrated that pancreatic MMP-9 was upregulated, and serum MMP-9 was elevated during caerulein-induced pancreatitis. Inhibition of MMP with BB-94 protected against pancreatic histological damage and reduced serum amylase and lipase. We further characterized the impact of MMP inhibition on pancreatic inflammatory infiltration and showed that inhibition of MMP-9 with BB-94 markedly reduced pancreatic neutrophil infiltration. Furthermore, inhibition of MMP-9 significantly downregulated neutrophil and macrophage-specific chemokines in the pancreas, leading to a reduction in the expression of proinflammatory cytokines and the central proinflammatory signal NF-*κ*B activation, which is likely mediated through inhibiting neutrophil ROS production and inflammatory macrophage polarization.

The critical role of the MMPs family in mediating inflammatory responses has been implicated in various inflammatory diseases, including AP [14, 31, 32]. The majority of the published studies on MMPs in AP were focused on their crucial role in regulating systemic inflammation and pulmonary complication associated with severe acute pancreatitis [12, 13, 16–18]. Limited studies showed the impact of MMPs, specifically MMP-9 on pancreatic inflammatory responses [14, 17]. Here, we showed that the mRNA level of MMP-9, but not MMP-2, was upregulated in caerulein-induced pancreatitis (data not shown), suggesting that MMP-9 plays a more predominant role during acute pancreatitis. Interestingly, inhibition of MMP with BB-94 markedly reduced pancreatic inflammatory infiltration, which is mediated by downregulating the expression of neutrophil and macrophage-relevant chemokines and the activation of the central proinflammatory signal NF-*κ*B, leading to the expression of proinflammatory cytokines. Scannevin et al. reported a highly selective chemical inhibitor of MMP-9, JNJ0966, which is an interesting potential candidate for testing in the acute pancreatitis model in the future [33].

Neutrophil infiltration occurs at the early stage of acute pancreatitis and plays a critical role in the development of AP [26]. Gukovskaya et al. showed that infiltrated neutrophils mediated pancreatic tissue damage via NADPH oxidase-mediated ROS production [34]. Further studies showed that neutrophil MMP-9 promotes neutrophil migration, pancreatic trypsinogen activation, and pancreatitis-associated lung injury *in vivo* [12, 14]. In this study, using isolated BMDNs, we found that inhibition of MMP with BB-94 significantly decreased neutrophil ROS production, suggesting the reduction in pancreatitis-associated tissue damages with BB-94 could be due to reduced neutrophil ROS production.

In AP, monocytes and macrophages infiltrated into the pancreas, differentiated into inflammatory M1-polarized macrophages, and mediated further tissue damage [35, 36]. Using BMDMs, we found that BB-94 inhibited M1 macrophage polarization. Furthermore, NF-*κ*B activation contributes to sustaining the status of M1-polarized macrophages, resulting in cytotoxic and inflammatory functions [30]. We showed that inhibition of MMP with BB-94 markedly reduced NF-*κ*B activation in M1-polarized macrophages. Collectively, these results showed that MMPs play a critical role in mediating inflammatory macrophage polarization, which in turn contributes to mediating further pancreatic inflammation and damage in AP.

In summary, our findings showed that inhibition of MMP with BB-94 protected against pancreatic inflammatory responses through inhibiting pancreatic inflammatory infiltration. The mechanism of this effect is likely via inhibiting neutrophil ROS production and inflammatory macrophage polarization. Our data suggest that targeting MMPs, particularly MMP-9, is a potential therapeutic approach for treating AP.

## Figures and Tables

**Table 1:** PCR genes primers sequences.

Gene (mouse)	Primer sequences
MMP-9 forward	5′-AGACGACATAGACGGCATCC-3′
Reverse	5′-TGGGACACATAGTGGGAGGT-3′
CCL2 forward	5′-AGACGACATAGACGGCATCC-3′
Reverse	5′-TGGGACACATAGTGGGAGGT-3′
CXCL2 forward	5′-CGCCCAGACAGAAGTCATAG-3′
Reverse	5′-TCCTCCTTTCCAGGTCAGTTA-3′
iNOS forward	5′-AGGGAATCTTGGAGCGAGTT-3′
Reverse	5′-GCAGCCTCTTGTCTTTGACC-3′
TNF-*α* forward	5′-TCTCTTCAAGGGACAAGGCTG-3′
Reverse	5′-ATAGCAAATCGGCTGACGGT-3′
IL-1*β* forward	5′-TTGACGGACCCCAAAAGAT-3′
Reverse	5′-GAAGCTGGATGCTCTCATCTG-3′
IL-6 forward	5′-TTCATTCTCTTTGCTCTTGAATTAGA-3′
Reverse	5′-GTCTGACCTTTAGCTTCAAATCCT-3′
*β*-Actin forward	5′-GTCCCTCACCCTCCCAAAAG-3′
Reverse	5′-GCTCCCTCAACACCTCAACCC-3′

## Data Availability

The data used to support the findings of this study are available from the corresponding author upon request.

## Abbreviations

AP: Acute pancreatitis
BMDNs: Bone marrow-derived neutrophils
BMDMs: Bone marrow-derived macrophages
CCL2: C-C motif chemokine ligand 2
CER: Caerulein
CXCL2: C-X-C motif chemokine ligand 2
MMP: Matrix metalloproteinase
PACs: Pancreatic acinar cells
PMA: Phorbol 12-myristate 13-acetate
ROS: Reactive oxygen species.
